# Improving educational quality through digital empowerment: a web platform for academic management in secondary education

**DOI:** 10.12688/f1000research.178595.1

**Published:** 2026-03-13

**Authors:** Lorena Soto Zenteno, Carlos Cayahuallpa, Alex Pacheco

**Affiliations:** 1 Professional School of Systems Engineering, Universidad Nacional de Cañete, San Vicente de Cañete, Lima, 15701, Peru

**Keywords:** web application, grade management, RUP, cross-curricular skills, secondary education, academic management.

## Abstract

**Background:**

Grade management in Peruvian educational institutions faces significant challenges due to the widespread use of Excel spreadsheets, which often lead to delays, errors, and fragmented information during registration, consultation, and report card preparation processes. National studies indicate that implementing web-based systems can reduce procedural times by 50% to 80%, improving efficiency and collaboration among teachers and administrative staff. In this context, the present study aims to improve grade management at Colegio Nacional de Imperial through the development of a web-based application designed to enhance accuracy, speed, and reliability in academic management.

**Methods:**

The system was developed using the Rational Unified Process (RUP), structured into four phases: inception, elaboration, construction, and transition. Requirements were analyzed, the system was designed and implemented using a relational database, and functionality was validated through testing. A quantitative pre-experimental design with pretest and posttest measurements was applied to evaluate performance improvements before full deployment.

**Results:**

The implementation of the web system produced substantial improvements in key grade management processes. The Grade Registration Time (GRT) decreased by 70.14% (from 4,080 to 864 seconds). The Grade Search Time (GST) improved by 79.39%, increasing efficiency and precision in data retrieval. The Report Card Generation Time (RCGT) showed the most significant reduction, decreasing by 97.74% (from 434.5 to 9.83 seconds), considerably streamlining report generation and improving access to academic performance information.

**Conclusions:**

The study demonstrates that a RUP-based web application significantly optimizes grade management in a rural secondary education context. The quantitative pre-experimental results confirm notable time reductions and improved information quality, highlighting the transformative potential of digital solutions in strengthening academic management processes in schools.

## Introduction

Currently, Basic Regular Education (EBR) institutions carry out various academic management tasks such as schedule planning, student enrollment, attendance, grade recording, and the delivery of learning progress reports known as report cards or grade reports. Teachers in EBR institutions have as one of their main functions the evaluation of activities and the recording of students’ grades during each bimester of the school year ( Ministerio de Educación, 2023). These activities are carried out in digital documents or, in some cases, manually using printed sheets, which generates greater effort and time consumption, negatively affecting the quality of processes and the level of satisfaction of the people involved ( Carbajal, 2018).

**Figure 1.**
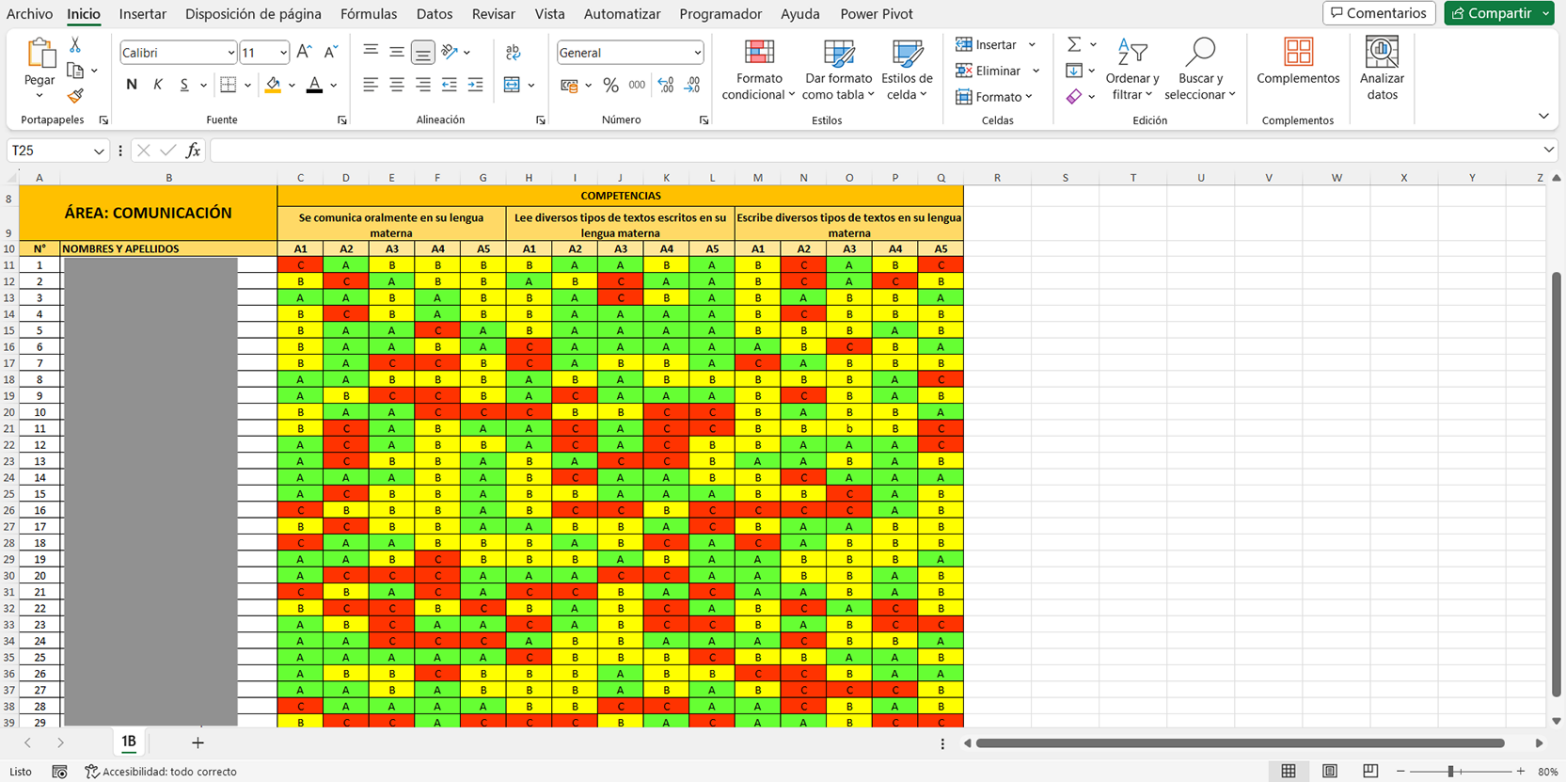
Grade recording before implementation. Source: Own elaboration.

The traditional grade recording process before system implementation is illustrated in Figure 1.

Students’ grade records are delivered to the school administrators so that they can be entered into the Educational Institution Management Support Information System (SIAGIE), which allows EBR institutions to carry out academic management tasks ( SIAGIE, 2023). However, as it is a nationwide platform, it experiences considerable slowness and, in some cases, system crashes due to the overwhelming number of users and data being entered, which prevents school administrators from obtaining reports on time. This generates the need to have their own system that optimizes these processes and guarantees the timely delivery of academic reports, as well as the obtaining of statistical data that SIAGIE does not provide.

Given these situations, the use of information technologies becomes an essential and indispensable tool to optimize the various processes of different institutions, companies, and organizations ( Flores-Chacón et al., 2023). In this same context, Basic Regular Education (EBR) institutions have not been exempt from the changes generated by the use of technologies in various areas of society ( Granados et al., 2020).

Peruvian researchers, ( Esteban & Trujillo, 2021) indicated that at I.E.P Nuestro Salvador in Villa María de Triunfo, Lima, grade recording was carried out manually using paper sheets and Excel spreadsheets, which caused various problems such as errors in grade entry, duplication of information, and loss of academic records. Therefore, the educational institution sought to solve these problems through the implementation of a web system.

Meanwhile, ( Angulo, 2021), with the implementation of his Web System to optimize the academic management process of I.E.P Niño Jesús de Belén, observed a notable improvement in the productive efficiency of the enrollment process, with an increase of 69.71%, and the level of compliance in the delivery of report cards improved by 50.89%.

In Trujillo, the implementation of an Integrated Web System to improve the academic management of E.S.F.A.P Macedonio de la Torre, ( Abad, 2020) achieved a significant reduction in the average times of different processes: enrollment registration was reduced to 9.45 minutes (equivalent to 70.16%); grade entry was reduced to 5.94 minutes (70.14%); the generation of grade reports was reduced to 3.17 minutes (70.14%); and the search for enrollments was reduced by 5.58 minutes (79.05%).

These difficulties are not only present in Peru, as this obstacle is reflected in the research work carried out by ( Pozo, 2023) in Ecuador, which indicates that the Unidad Educativa Ancón performed grade recording manually or using digital documents because, although they had a system for these processes, it did not cover all needs and had many shortcomings. Therefore, the institution found it necessary to have a web application, where after its implementation the result was a reduction in grade entry time and report generation by 80%.

Finally, in Colombia, ( Ibañez, 2021) integrated the Automation of student grade recording into a web platform, for which 5 phases were carried out in the development of the project: (1) Understanding of the current process, (2) Form creation, (3) System coding, and (4) Automation and system quality testing. The final result was a reduction in grade recording time from 50 to 2 minutes; that is, there was a 96% improvement in performing this task.

## Methods

The web application was developed following the phases and disciplines defined in the Rational Unified Process (RUP) methodology. The activities performed throughout the development lifecycle and the corresponding deliverables are described in this section. The phases, disciplines, and deliverables involved in the development process are summarized in Table 1.

**Table 1.** Phases, disciplines, and deliverables for the development of the web application.

Phases	Disciplines	Deliverables
Inception	Business process modeling	Modeling of the grade control process
		Software development plan
Elaboration	Requirements	Requirements gathering
		Identification and description of functional and non-functional requirements
		Prioritization of functional requirements
	Análisis y diseño	Modeling of functional requirements (use cases and specification)
		Requirements acceptance document
		Software architecture design
		Database design
Construction	Transition	Coding of components
Transition	Deployment	Launch of the web application Web application delivery report

Source: Own elaboration.

### • First phase: Inception

In this phase of the project, the goal was to describe the initial case of the educational institution through a flowchart to determine whether the grade control web application would represent significant value. Therefore, to begin this phase, a meeting was first held with the administrators of I.E.P “Imperial,” where the problem to be solved and the technological solution to be implemented were presented. Since benefits and improvements that the implementation would bring to the grade control process were identified, the project was accepted and, consequently, the software development began.

**Figure 2.**
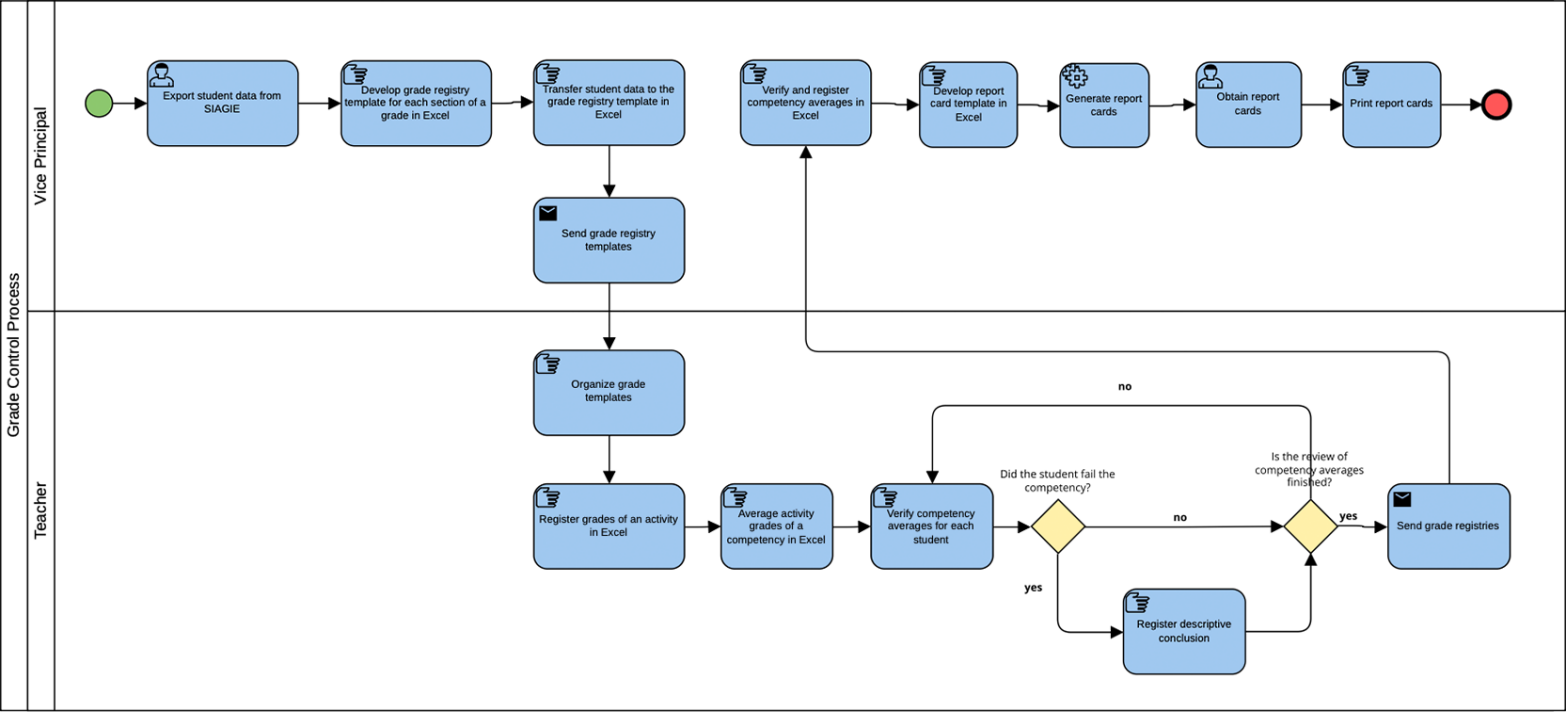
Modeling of the grade control process (flowchart). Source: Own elaboration.

The modeling of the grade control process is shown in Figure 2.

### • Second phase: Elaboration

**Figure 3.**
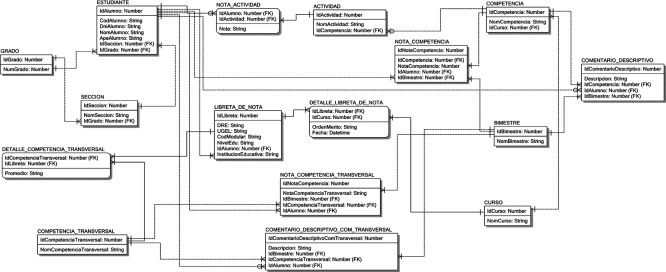
Logical design of the web application database. Source: Own elaboration.

**Figure 4.**
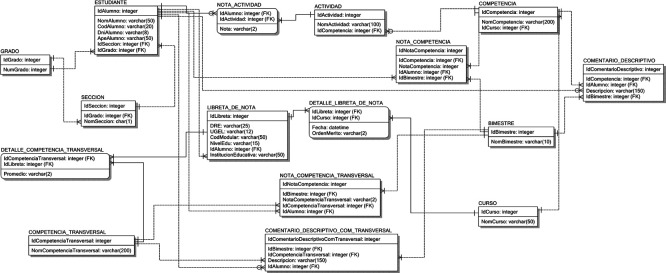
Physical design of the web application database. Source: Own elaboration.

During this phase, the initial user requirements were detailed, and both the functional and non-functional requirements of the system were described and evaluated. The detailed user requirements identified during the analysis phase are summarized in Table 2. Subsequently, the functional requirements were modeled to support the architectural design of the system, and the database structure of the web application was developed. The logical database design is illustrated in Figure 3, while the physical database design is presented in Figure 4.

**Table 2.** User requirements gathering.

Techniques	Description of user requirements		
	Teachers	Vice principal	Principal
Documentary Analysis	Teachers need to register the activities evaluated in each competency of a course and for each bimester.	The vice principal needs to register the courses.	The principal needs to view the students’ grade records for each section.
	Teachers need to enter the grades of evaluated activities for students according to the competency of a course and for each bimester. The grading scale is as follows: AD = outstanding achievement A = achieved B = in progress C = beginning SN = no grade NST = did not work on the competency	The vice principal needs to register the competencies of a course.	The principal needs to view the records of transversal competency grades of students in each section.
	Teachers need to enter the grades of transversal competencies of each student and for each bimester. The grading scale is as follows: AD = outstanding achievement A = achieved B = in progress C = beginning NT = no grade NST = did not work on the competency	The vice principal needs to register the grade levels.	The principal needs to view the students’ report cards for each section.
		The vice principal needs to register the sections of a grade level.	The principal needs to view the merit order of students in each section.
		The vice principal needs to assign teachers to a section of a grade level and to a course.	
		The vice principal needs to assign teachers as tutors of a section of a grade level.	
		The vice principal needs to register students according to their grade level and section.	
		The vice principal needs to register transversal competencies.	
		The vice principal needs to prepare the report card of each student and for each bimester. It must display the educational institution’s information, student data such as grades obtained in each course competency, justified and unjustified absences and tardiness; and the teacher’s descriptive conclusions. Additionally, the system must allow storing, modifying, deleting, and printing the reports.	
		The vice principal needs to view the grades of students’ competencies.	
		The vice principal needs to view the grades of students’ transversal competencies.	
Interview	Teachers need to enter a descriptive conclusion for each course competency and for each bimester.	The vice principal needs to obtain the merit order report of students in each section of a grade level for each bimester.	
	Teachers need to enter a descriptive conclusion for each transversal competency evaluated for a student and for each bimester.		

Source: Own elaboration.

This phase was essential as it ensured that the system was developed on a solid foundation, since what is detailed in this phase is what was implemented in the web application.

### • Third phase: Construction

*Development Technologies and Add-ons*

The programming language used was PHP, selected for its ease of use, its wide adoption in web application development, and the large variety of available libraries that simplify programming tasks. This choice aligns with similar educational systems, such as the one developed by ( Oscco, 2022), which highlights its simplicity and scalability for educational environments.

*Customization for Educational Institutions*

The software was specifically adapted to the needs of Peruvian educational institutions, customizing data categorization, user roles, and report generation for more effective grade management. This practice is reflected in the research of ( De La Cruz et al., 2025), where academic monitoring modules were adjusted to tutor and student roles, improving efficiency in personalized processes. Likewise, ( Huaman-Yupanqui et al., 2024) implemented a web system with Scrum at the Universidad Nacional Amazónica de Madre de Dios, adapting user registration, enrollment, and certificate functions to the institution’s local regulations.

*Technological details for indexing consideration*

▪ Backend Framework: The system runs on PHP 8.1.
▪ Frontend Framework: The user interface is developed with Bootstrap, an open-source framework recognized for its responsive design adaptable to different devices.
▪ Add-ons and libraries: Resources such as TCPDF were strategically integrated for the generation of report cards in PDF format and ApexCharts to provide the system with statistical chart visualization capabilities, in addition to other libraries that contribute to improving the user experience.

### • Fourth phase: Transition

In this phase, it was ensured that the software was ready for end users, and all aspects necessary for a successful implementation were managed.

**Deployment**

At this stage, the complete and functional version of the system is launched in the production environment, verifying that all functionalities are operational and that the application meets the requirements defined by the institution. In this way, the system is ready for real use by teachers and administrative staff.

Minimum system requirements:

▪ Server:
  • A server environment compatible with PHP 8.1.
  • Sufficient storage capacity to manage institutional information, using a MySQL database.
▪ Client:
  • A modern web browser with JavaScript enabled.
  • An Internet connection to allow access to the web-based system.

By ensuring compliance with these requirements, it is guaranteed that the software tool remains accessible and functional even in educational environments with limited resources, promoting its adoption and continuous use.

## Use Cases

In this section, the results obtained regarding the grade management process and the user experience with the platform are presented.

### Use Case 1: Grade Registration

**Figure 5.**
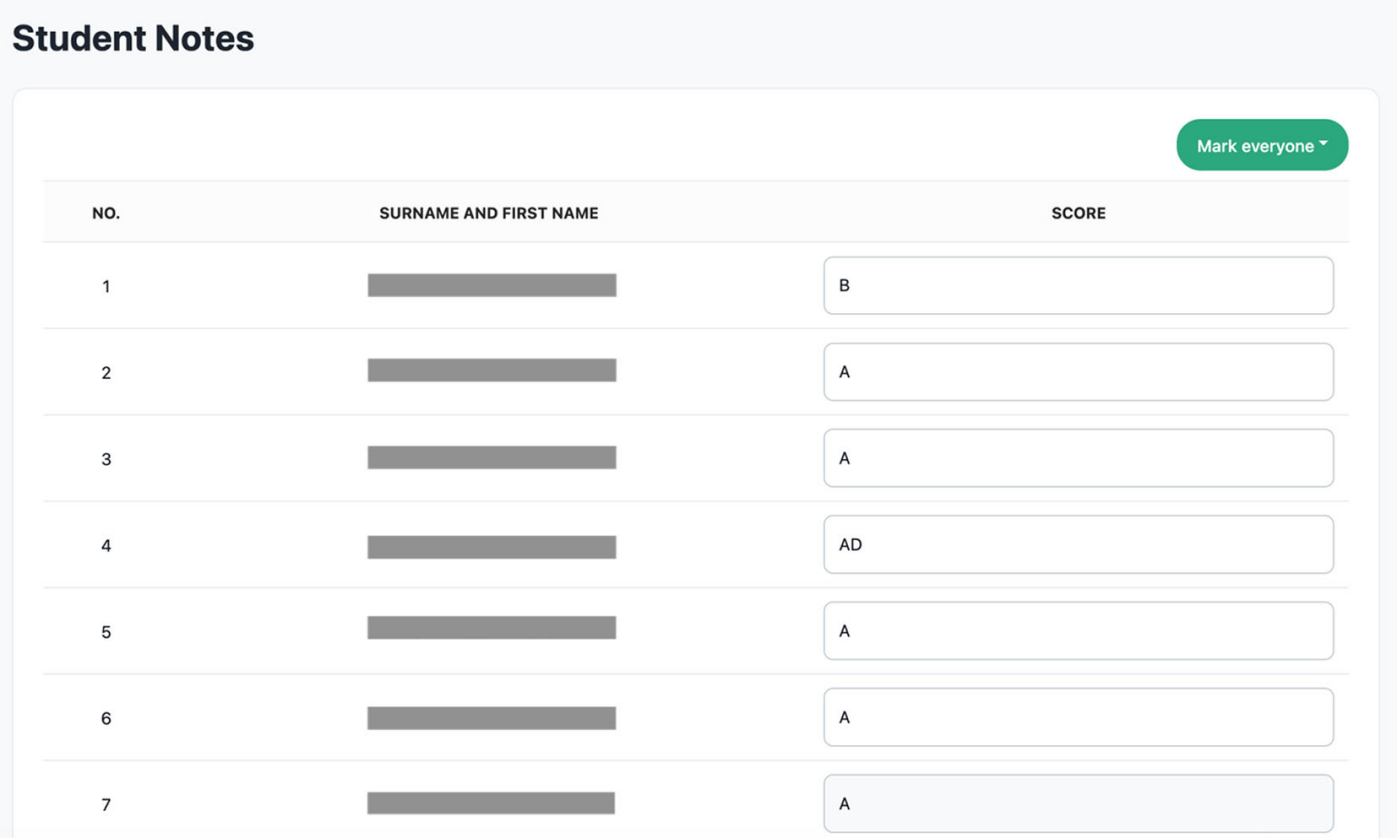
Grade registration. Source: Own elaboration.

To verify the functionality of the software, a use case based on the grade registration process within the software is presented. In this case, the teacher accesses the grades module and proceeds to record the evaluations of each student, previously selecting the course, grade level, section, and corresponding activity. The system facilitates the orderly entry of information, validates the data entered, and securely stores it in the database, optimizing teaching tasks and ensuring precise control of academic information, as shown in Figure 5. This flow coincides with the use case described by ( León-Velarde et al., 2024), where the teacher records grades by subject, partial term, and bimester in an ASP.NET web system, validating data and automatically generating a history for students.

**Input:**

N: Number in the list, ordered by surname

Fullname: Surname and Firstname, no editable

SCORE: AD, A, B, C or NT, editable

### Use Case 2: Grade Search

**Figure 6.**
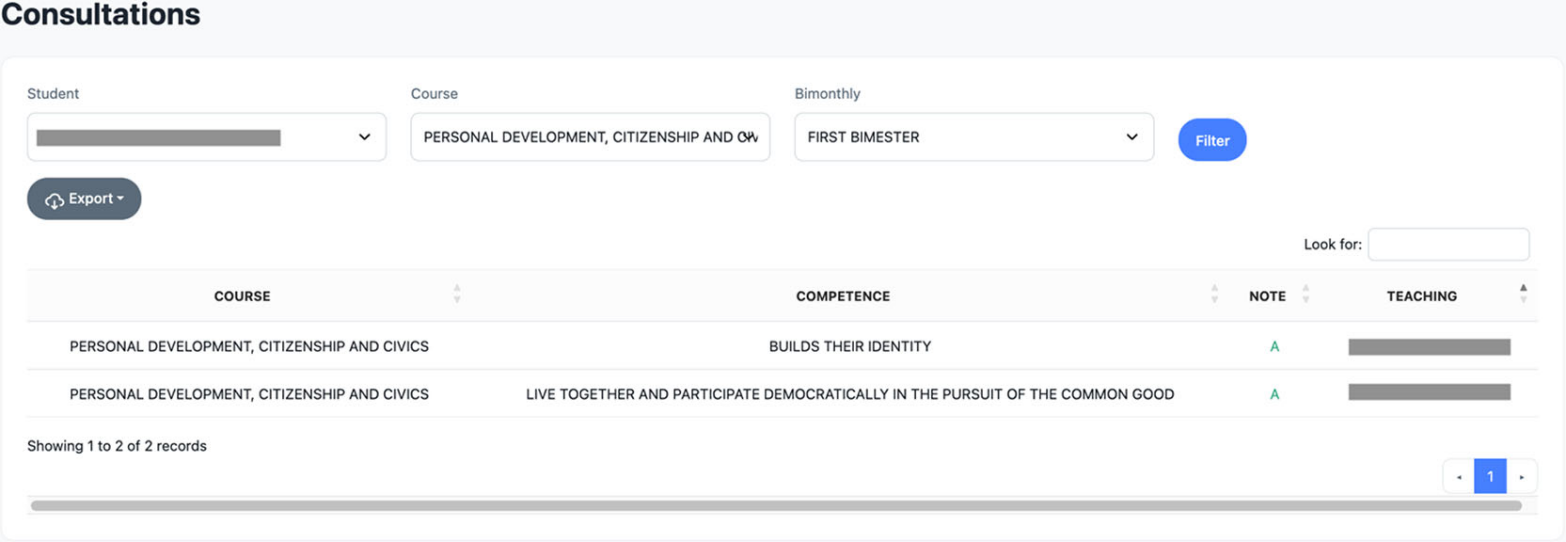
Grade search. Source: Own elaboration.

To demonstrate the functionality of the software, a use case focused on searching for grades within the academic system of the educational institution is presented. In this scenario, the user performs a query by entering specific criteria such as the student’s name, course, and academic period. When executing the search, the software processes the request and displays a list of results that match the established parameters. This practical case demonstrates the system’s ability to facilitate the rapid and accurate retrieval of relevant academic information for teaching and administrative staff, optimizing grade management and consultation, as shown in Figure 6. This flow aligns with the search use case in the research by ( Ramos-Miller & Pacheco, 2023), where the user enters criteria such as asset name, category, or location in a web system, processing the query and displaying a list of matches with details such as description and status.

**Input:**

Filters applied in the form:

Student: XXXX

Course: PERSONAL DEVELOPMENT, CITIZENSHIP AND CIVICS

Bimonthly: FIRST BIMESTER

**Output:** List of coincidences:

• PERSONAL DEVELOPMENT, CITIZENSHIP AND CIVICS
  ○ Competence, Grade, Teacher

### Use Case 3: Generation of Report Cards According to UGEL Guidelines

**Figure 7.**
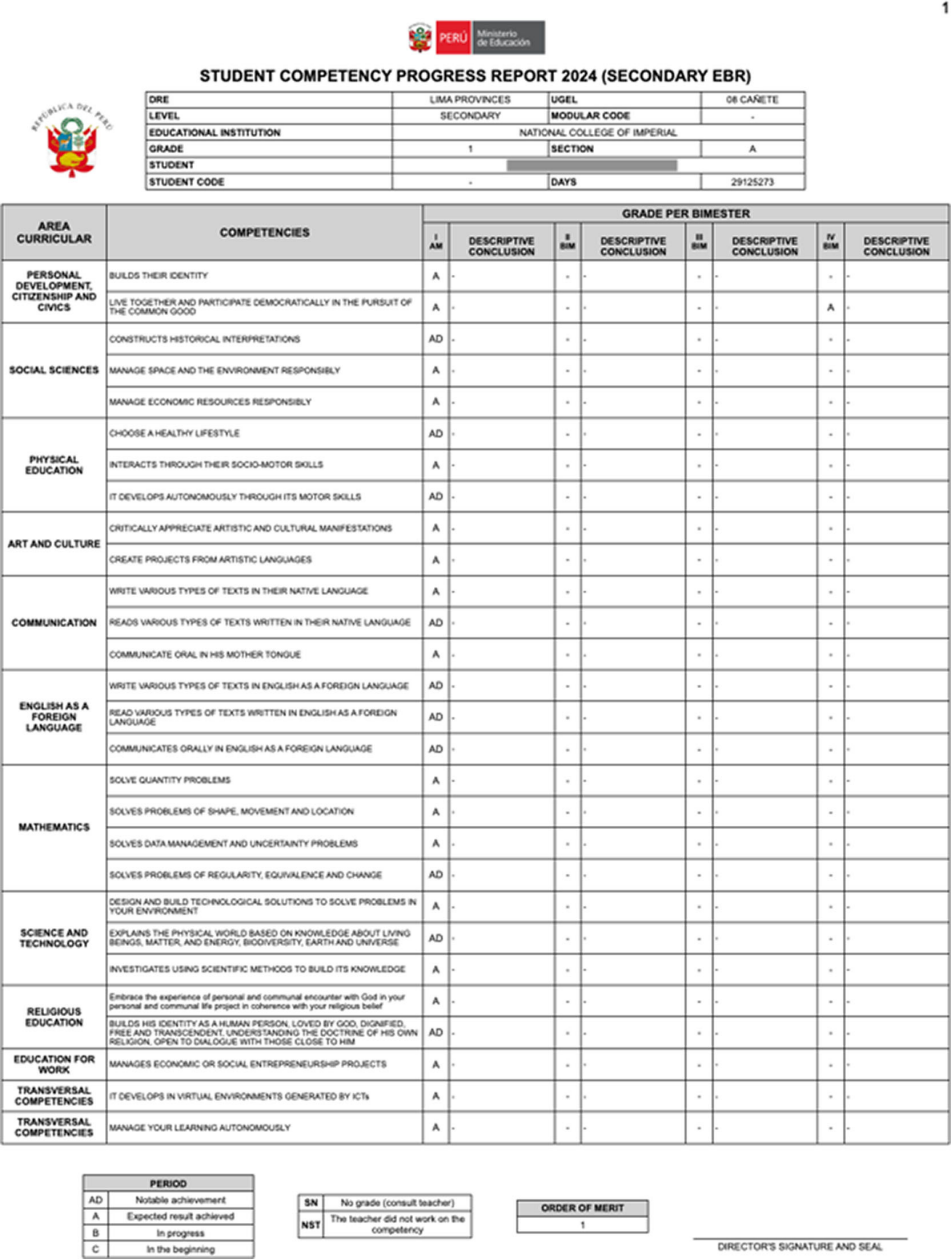
Report cards generated by the software. Source: Own elaboration.

This scenario demonstrates the functionality of the software for the automatic generation of report cards in accordance with the guidelines established by the Local Educational Management Unit (UGEL). The user accesses the reports module, where options can be selected to generate report cards by student, by classroom, by grade level, or by academic period. Based on this selection, the system processes the recorded information, organizes the grade data, and presents it in a standardized format that complies with the official UGEL requirements. This functionality allows the educational institution to efficiently prepare and issue formal academic documents, ensuring regulatory compliance and facilitating administrative management, as shown in Figure 7.

**Input:**

• Access the “Progress Report” view
**Output:**
• Progress Report per student

## Discussion

The findings of the research show that the grade registration time before the implementation of the web application was 4080 seconds, whereas with the implementation of the web application the time was reduced to 864 seconds, achieving an improvement of 79.39% in the grade registration time indicator. This result is consistent with ( Ramadhani et al., 2025), who developed a web system that integrates the recording of daily, midterm, final grades, and attendance with automatic calculation of the final grade, eliminating manual entry errors and slow processes typical of manual school management. Likewise, ( Amfotis & Wati, 2025) optimized grade registration through a web system that automates the complete flow of master data and reports, improving overall efficiency in educational environments.

Regarding the second indicator, the results show that the grade search time before the implementation of the web application was 789 seconds, while with the implementation of the web application the time was 162.63 seconds, achieving a significant improvement of 79.39% in the grade search time indicator. This result is contrasted with ( Ugalingan & Antonio, 2025), who through quasi-experimental tests demonstrated greater speed and accuracy in searching academic records when comparing the manual system with the automated web version. Similarly, ( Patil et al., 2023) facilitated rapid searches of student records thanks to their web structure with access roles, reducing time compared to traditional methods.

Finally, regarding the third indicator, the research results show that the time required to prepare report cards before the implementation of the web application was 434.5 seconds, whereas with the implementation of the web application the time was reduced to 9.83 seconds, indicating a 97.74% improvement in the report card preparation time indicator. This progress is consistent with ( Nitron, 2024), who integrated the automatic generation of grade reports and report cards, streamlining administrative processes that were previously manual and prone to duplication. Likewise, ( Patiam, 2024) optimized the preparation of grade reports and class records through web automation, focusing on reducing teachers’ workload in repetitive activities.

## Conclusions

It is determined that the web application significantly improves the grade control of students at Colegio Nacional de Imperial. This is supported by the results obtained in the indicators TRN, TBN, and TELN, which, after being subjected to testing, demonstrated a positive effect following the implementation of the web application.

A significant improvement of 70.14% was obtained in the grade registration time (TRN) of student grade control. This result determines the fulfillment of the first specific objective proposed, demonstrating the effectiveness of the implemented web application in optimizing and streamlining the grade registration process.

An improvement of 79.39% was obtained in the grade search time (TBN) of student grade control at Colegio Nacional de Imperial. This result determines the fulfillment of the second specific objective proposed, demonstrating the effectiveness of the implemented web application in accelerating grade consultation, facilitating faster and more efficient access to information.

A significant improvement of 97.74% was obtained in the report card preparation time (TELN) of student grade control at Colegio Nacional de Imperial, Cañete, 2023. This result determines the fulfillment of the third specific objective proposed, demonstrating the effectiveness of the implemented web application for generating grade reports, making the process faster and more accurate, which has allowed the timely preparation and delivery of reports.

## Ethics and consent

Ethics and consent are not required for this article.

## Software availability

DIGIEDU: A Web-Based Grade Management System

• Source code available from: https://github.com/pKroz/DIGIEDU
• Archived software available from: https://doi.org/10.5281/zenodo.18494715 ( Soto Zenteno, 2026b)
• License: MIT License.

## Data Availability

Zenodo: Pretest and posttest – Grade Management variable, https://doi.org/10.5281/zenodo.18492650 ( Soto Zenteno, 2026a). This project contains the following data:
• DATA Record Sheets - DIGIEDU.xlsx Data are available under the terms of the Creative Commons Attribution 4.0 International license (CC-BY 4.0).
