# Supplementary material for: Improving educational quality through digital empowerment: a web platform for academic management in secondary education
Source: F1000Res. 2026 Mar 13;15:402. [Version 1] doi: 10.12688/f1000research.178595.1 (PMC13270190; doi:10.12688/f1000research.178595.1)
Supplement: Peer review history

# Reviewer report: Reviewer response for version 1

**DOI:** 10.5256/f1000research.197001.r480911

**Published:** 2026-06-15

**Authors:** Irwansyah Irwansyah

**Affiliations:** 1 Universitas Indonesia, Depok, West Java, Indonesia

**Overall assessment**

 This article presents DIGIEDU, a web-based academic management system developed to improve grade registration, grade searching, and report-card generation at Colegio Nacional de Imperial in Peru. The topic is practically important, particularly for schools that continue to rely on spreadsheets, manually transferred records, or national systems that may be slow or unable to produce locally required reports. The manuscript provides a generally coherent account of the institutional problem, the Rational Unified Process used for software development, the principal user requirements, the database design, the technologies used, and several representative workflows.

 The software appears useful and the reported reductions in task-completion times are substantial. The availability of the source code, archived software, and evaluation dataset is also a strength. However, the article does not yet provide sufficient methodological and technical information to permit independent replication of either the software deployment or the performance evaluation. There are also unresolved numerical inconsistencies, unsupported claims about significance, accuracy, information quality, and educational quality, and an insufficiently justified statement that ethics approval and consent were not required.

 My overall recommendation is **approved with reservations**, requiring major revisions focused on reproducibility, analytical transparency, data governance, and alignment between the evidence and the conclusions.

**Summary of the article**

 The article addresses inefficiencies in academic management within a Peruvian secondary school. The existing process reportedly depended on Excel spreadsheets, printed records, manual transfers, and the national Educational Institution Management Support Information System, known as SIAGIE. The authors argue that this arrangement creates delays, fragmented information, data-entry risks, and difficulties in producing timely academic reports.

 To address these problems, the authors developed DIGIEDU using the Rational Unified Process. The development process is organised into inception, elaboration, construction, and transition phases. Requirements were gathered through documentary analysis and interviews with users occupying teacher, vice-principal, and principal roles. The application supports grade entry, searches for academic records, generation of report cards, management of courses and competencies, student registration, and production of merit-order reports.

 The reported technology stack includes PHP 8.1, Bootstrap, MySQL, TCPDF for PDF generation, and ApexCharts for visualisation. The article includes process diagrams, logical and physical database diagrams, user requirements, screenshots, and three principal use cases.

 The authors evaluate performance through a pretest and posttest design. They report substantial reductions in the time needed to register grades, search for grades, and generate report cards. The source code is available through GitHub and an archived Zenodo record, while the pretest and posttest data are also deposited in Zenodo.

 The tool has clear practical potential. Nevertheless, the current presentation does not allow readers to determine precisely how the performance measurements were obtained, how many observations or users were included, whether the reported differences were statistically tested, or whether the published code can reproduce the evaluated version.

**Responses to the review questions**

 1. Is the rationale for developing the new software tool clearly explained?

**Yes.**

 The manuscript clearly identifies the practical problem. Existing grade-management activities depend on spreadsheets, printed documents, manual data transfer, and a national platform that is described as slow or insufficient for local reporting needs. The authors connect these limitations to delays, duplicated work, errors, fragmented information, and difficulties producing report cards and statistical summaries.

 The manuscript also explains why an institution-specific web system may be useful. The proposed tool is intended to support locally required grade categories, role-based workflows, academic competencies, transversal competencies, merit-order reports, and report cards compliant with Local Educational Management Unit requirements.

 The rationale could nevertheless be strengthened by explaining more explicitly:

1. Why adapting or extending SIAGIE was not feasible.
2. Whether DIGIEDU complements or replaces any SIAGIE functions.
3. What is technically or functionally novel compared with the many similar grade-management systems cited in the introduction.
4. Which requirements are specific to the Peruvian competency-based grading system.
5. Whether interoperability with national education systems is planned.

These additions would sharpen the contribution but are not essential to establishing the basic rationale.

 2. Is the description of the software tool technically sound?

**Yes, at a general level.**

 The software description is coherent enough to understand its intended operation. The article identifies user roles, functional requirements, database entities, principal technologies, minimum server and client requirements, deployment context, and representative workflows. Logical and physical database diagrams are provided, and screenshots demonstrate grade registration, grade searching, and report-card generation.

 Several technical points nevertheless require clarification.

 First, PHP 8.1 is described as a backend framework. PHP is a programming language and runtime environment, not necessarily a framework. The authors should identify the actual framework, if one was used, or state that the software was developed in plain PHP.

 Second, the manuscript does not provide a system architecture diagram showing the relationships among the browser interface, application layer, authentication layer, database, PDF-generation service, and visualisation components.

 Third, important technical safeguards are not described. Because the tool processes the records of school students, including minors, the manuscript should explain:

- authentication and password-storage procedures;
- role-based authorisation;
- session management;
- protection against SQL injection, cross-site scripting, and cross-site request forgery;
- server-side validation of grades and user input;
- encrypted connections;
- database backup and recovery;
- audit logs;
- data retention and deletion procedures;
- access restrictions for sensitive student records.

The absence of these details does not demonstrate that the software is technically unsound, but it prevents a complete evaluation of its reliability, security, and suitability for real institutional deployment.

 3. Are sufficient details of the code, methods, and analysis provided to allow replication of the software development and its use by others?

**Partly.**

 The article provides useful high-level materials, including the software-development method, user requirements, database diagrams, technologies, use cases, source-code repository, archived software, and minimum system requirements. These materials provide a starting point for replication.

 They are not sufficient for an independent researcher or school administrator to reproduce the evaluated system reliably. The following information is required.

 Software version and repository linkage

 The authors must:

1. Identify the exact release, commit hash, or version tag used in the study.
2. Confirm that the Zenodo archive corresponds exactly to the evaluated version.
3. Explain the relationship between the GitHub repository, Zenodo archive, and manuscript.
4. Provide a software citation containing the version and permanent identifier.
5. State whether later repository changes affect the reported results.

Installation and configuration

 A reproducible installation guide should include:

1. Supported operating systems.
2. Web-server requirements, such as Apache or Nginx and their versions.
3. Exact PHP, MySQL, Bootstrap, TCPDF, ApexCharts, and other dependency versions.
4. Required PHP extensions.
5. Database creation and migration instructions.
6. Configuration-file and environment-variable requirements.
7. A safe example configuration without passwords or credentials.
8. Procedures for creating the first administrator account.
9. Instructions for importing demonstration or seed data.
10. Commands for running the application and automated tests.

A containerised deployment, such as Docker, would be helpful but is not mandatory if complete manual installation instructions are supplied.

 Architecture and implementation logic

 The authors should provide:

- a system architecture diagram;
- a module description;
- a permissions matrix for each user role;
- database schema tables listing fields, data types, keys, constraints, and relationships;
- descriptions or pseudocode for grade validation, report-card generation, merit-order calculation, searching, and authentication;
- explanations of how missing grades, repeated records, invalid grade symbols, and conflicting updates are handled;
- details of functional, integration, security, and user-acceptance testing.

Replication of the performance evaluation

 The manuscript must explain how another researcher can reproduce the reported timing results. The current article does not clearly specify:

- the number of participating users;
- the number of observations or repeated trials;
- whether the same users completed pretest and posttest tasks;
- the number of students, classes, courses, and records processed;
- whether test data or real institutional data were used;
- the hardware, operating system, server, browser, and network conditions;
- when timing began and ended;
- whether loading time, data entry, verification, and printing were all included;
- whether users received training before the posttest;
- who recorded the times and with what instrument;
- how failed attempts, interruptions, outliers, and missing values were treated;
- whether task order was standardised;
- whether the pretest and posttest used tasks of equivalent complexity.

Without these details, the reported evaluation cannot be independently replicated.

 4. Is sufficient information provided to allow interpretation of the expected output datasets and results generated using the tool?

**Partly.**

 The article makes the main functional outputs understandable. Readers can identify outputs such as recorded grades, filtered search results, competency reports, report cards, and merit-order reports. Screenshots and use cases also illustrate what users are expected to enter and receive.

 The quantitative evaluation dataset is less adequately documented. The authors should add a data dictionary explaining:

- the meaning of every variable and abbreviation;
- the unit of observation;
- whether each row represents a user, task, trial, class, or aggregated result;
- the meaning of pretest and posttest;
- units of measurement;
- permitted values and coding rules;
- missing-value codes;
- anonymised participant identifiers;
- task categories;
- hardware or network conditions;
- success and error indicators;
- formulas used to calculate percentage reductions.

A table in the manuscript should link each reported indicator directly to the relevant dataset columns. The authors should also provide a short worked example showing how one reported percentage was calculated from the underlying observations.

 The grade categories are described, but abbreviations such as SN, NT, and NST appear to be used inconsistently. These codes should be standardised and defined once. The English abbreviations used in the abstract and the Spanish-derived abbreviations used in the conclusions should also be harmonised.

 5. Are the conclusions about the tool and its performance adequately supported by the findings?

**Partly.**

 The descriptive results support the limited conclusion that the web application was associated with faster completion of three administrative tasks in the evaluated setting. The reductions reported for grade searching and report-card generation are substantial.

 The broader conclusions are not fully supported for five reasons.

 Numerical inconsistency

 The grade-registration result is reported inconsistently:

- the abstract and conclusion state a reduction of 70.14%;
- the discussion states a reduction of 79.39%;
- the reported times are 4,080 seconds before implementation and 864 seconds after implementation.

Using those two reported times, the calculated reduction is approximately 78.82%, not 70.14% or 79.39%. The authors must return to the raw data, identify the correct values, explain the discrepancy, and correct the abstract, results, discussion, conclusion, tables, and dataset as necessary.

 Unsupported claims of statistical significance

 The manuscript repeatedly uses terms such as “significant improvement,” but it does not clearly report a statistical test, sample size, variability, confidence interval, or effect size. A percentage reduction alone does not establish statistical significance.

 If repeated paired observations are available, the authors should report appropriate paired analyses. Depending on the data distribution, this may involve a paired-samples test or a non-parametric alternative. The revised manuscript should include:

- sample size;
- descriptive statistics;
- standard deviations or interquartile ranges;
- confidence intervals;
- statistical test and assumptions;
- exact p-values;
- effect sizes.

If only one aggregated pretest and posttest value exists for each task, inferential claims must be removed. The findings should then be described as descriptive case-study results rather than statistically significant effects.

 Unsupported claims about accuracy and information quality

 The study measures time, but the conclusions also claim improved accuracy, precision, reliability, and information quality. No error rates, record-validation results, data-completeness measures, reliability tests, or comparisons with verified reference records are presented.

 The authors should either add evidence for these outcomes or remove the claims. Appropriate measures could include:

- frequency of data-entry errors;
- percentage of complete records;
- agreement with manually verified records;
- number of duplicate or invalid entries;
- report-generation failures;
- search precision or retrieval accuracy;
- system uptime and error logs.

Overstatement of educational impact

 The title and conclusion refer to improving educational quality and the transformative potential of digital solutions. The study measures administrative task-completion time, not learning outcomes, teaching quality, student achievement, user satisfaction, or institutional decision quality.

 The authors should either:

1. narrow the title and conclusions to academic-management efficiency; or
2. add valid measures of educational or organisational quality.

The existing evidence supports improved administrative efficiency, not a general improvement in educational quality.

 Design limitations

 The evaluation uses a single-group pretest and posttest design at one institution. It does not include a control group, randomisation, multiple sites, long-term follow-up, or evidence that the changes were caused exclusively by the software.

 A limitations section should discuss:

- the single-institution setting;
- the absence of a control or comparison group;
- learning and practice effects;
- differences in hardware or network conditions;
- possible observer effects;
- limited generalisability;
- the short evaluation period;
- the absence of long-term maintenance and adoption data.

Points that must be addressed for scientific soundness

 The following revisions are essential.

 1. Reconstruct and fully report the evaluation method

 The authors must report the participants or testing agents, sample size, unit of analysis, number of observations, task definitions, timing protocol, equipment, network conditions, training, data-collection dates, and treatment of missing or anomalous observations.

 2. Correct all numerical inconsistencies

 The grade-registration figures must be recalculated from the raw data. All percentages, times, acronyms, tables, dataset entries, and narrative statements must agree.

 3. Provide defensible statistical analysis or remove inferential language

 Claims of significance require sample sizes, appropriate tests, uncertainty estimates, and effect sizes. If such analysis is impossible, “significant” must be replaced with accurate descriptive wording.

 4. Align the conclusions with the measured outcomes

 Claims about educational quality, accuracy, precision, reliability, information quality, and transformation must either be supported by corresponding evidence or removed. The conclusion should primarily concern administrative task efficiency unless additional outcomes are evaluated.

 5. Provide reproducible technical documentation

 The exact software version, architecture, dependencies, database setup, configuration, installation steps, role permissions, core logic, testing procedures, and instructions for reproducing the reported workflows must be supplied.

 6. Document the evaluation dataset

 The authors must provide a data dictionary, unit of observation, coding rules, formulas, missing-data procedures, and a direct correspondence between the deposited data and each reported result.

 7. Address security and privacy

 The revised article must explain how student records are protected, including authentication, authorisation, validation, secure storage, backups, auditability, and protection against common web vulnerabilities.

 8. Clarify ethics and consent

 The statement that ethics and consent were not required is insufficiently justified. The manuscript refers to interviews, institutional users, pretest and posttest activities, and student academic records.

 The authors must clarify whether:

- teachers, administrators, or other individuals participated in interviews or performance testing;
- real student records were used;
- data were identifiable;
- informed consent was obtained;
- an institutional ethics committee reviewed the work;
- a formal exemption or waiver was granted.

If the evaluation used only synthetic data and developer-operated tests, this should be stated explicitly. If human participants or identifiable student records were involved, the appropriate approval, exemption, consent, and data-protection information must be reported.

**Additional recommended revisions**

 The authors should also consider the following improvements:

1. Add a concise limitations section.
2. Distinguish clearly between software-development results and evaluation results.
3. Standardise the school’s name throughout the article.
4. Standardise all indicator names and abbreviations.
5. Clarify whether the setting meets a formal definition of rural education.
6. Improve the legibility of the database diagrams.
7. Replace comparisons with loosely related software systems with comparisons based on equivalent tasks and evaluation measures.
8. Explain how DIGIEDU interacts with SIAGIE and UGEL requirements.
9. Report usability or user-acceptance evidence if claims about user experience are retained.
10. Verify that the repository contains no credentials or identifiable student data.

**Final recommendation**

 The article addresses a real educational-administration problem and presents a potentially valuable open-source tool. Its rationale and high-level technical design are sufficiently clear, and the reported efficiency gains justify continued consideration. However, the current manuscript does not yet meet full standards of scientific reproducibility and analytical transparency.

 I recommend approved with reservations, subject to major revision. The authors must correct the numerical results, fully describe the evaluation method, justify or remove claims of statistical significance, align the conclusions with the measured evidence, document the dataset and software sufficiently for replication, and resolve the ethics, security, and privacy issues.

Are the conclusions about the tool and its performance adequately supported by the findings presented in the article?

Partly

Is the rationale for developing the new software tool clearly explained?

Yes

Is the description of the software tool technically sound?

Yes

Are sufficient details of the code, methods and analysis (if applicable) provided to allow replication of the software development and its use by others?

Partly

Is sufficient information provided to allow interpretation of the expected output datasets and any results generated using the tool?

Partly

Reviewer Expertise:

Educational technology; information systems; digital transformation in education; web-based academic management systems; software tools for school administration; quantitative evaluation of digital platforms.

I confirm that I have read this submission and believe that I have an appropriate level of expertise to confirm that it is of an acceptable scientific standard, however I have significant reservations, as outlined above.

# Reviewer report: Reviewer response for version 1

**DOI:** 10.5256/f1000research.197001.r480917

**Published:** 2026-06-15

**Authors:** Rubén Baena-Navarro

**Affiliations:** 1 Universidad de Córdoba, Montería, Colombia

Dear authors,

 Thank you for the opportunity to review the manuscript “Improving educational quality through digital empowerment: a web platform for academic management in secondary education”. The article addresses a relevant applied problem in secondary school academic administration: the continued reliance on spreadsheets, manual records, and fragmented procedures for grade registration, grade consultation, and student progress report generation. The main contribution of the work is the development and presentation of a web-based application, DIGIEDU, intended to optimize these processes at Colegio Nacional de Imperial through a RUP-based software development lifecycle and a pretest–posttest assessment of operational time reductions.

 Regarding the specific questions in the review form, I consider that the rationale for developing the software tool is clearly explained. The manuscript identifies a concrete institutional problem —delays, duplication of information, extensive use of Excel, and poorly integrated procedures— and proposes a technological solution aligned with that need. However, I answered “Partly” to the remaining questions because the technical description, reproducibility of the development process, interpretation of the results, and support for the conclusions require substantive clarification and correction.

 The manuscript has several real strengths. First, the problem addressed is verifiable and connected to a concrete operational need within the educational institution. Second, the use of the Rational Unified Process provides an understandable structure for the development lifecycle, organized into inception, elaboration, construction, and transition. Third, the inclusion of the previous grade-recording process, user requirements by role, logical and physical database designs, use cases, source code, archived software, and associated dataset are valuable elements for a Software Tool article. Fourth, the selected indicators —grade registration time, grade search time, and report card generation time— are relevant for assessing administrative efficiency in the context of the developed system.

 Nevertheless, several points must be addressed for the article to reach a sufficiently robust level of scientific soundness.

 The first essential correction concerns the numerical consistency of the reported results. The manuscript states that grade registration time decreased from 4,080 to 864 seconds. However, the reported percentage improvement is inconsistent: in some sections it is presented as 70.14%, whereas in another section it is reported as 79.39%. Using the standard percentage reduction formula, these values do not yield 70.14%. This inconsistency directly affects one of the central findings of the article. The authors should explicitly state the formula used to calculate each percentage improvement, verify the source data, and correct the abstract, discussion, conclusions, and any related table or dataset where the indicator appears.

 A second essential revision concerns the pretest–posttest measurement methodology. The article indicates that a quantitative pre-experimental design was applied, but it does not describe with sufficient precision how the reported times were obtained. The manuscript should specify the sample size, the number of users or participants, the number of records processed, the number of repetitions per task, the profile of the operators, the technical conditions of the environment, the timing instrument, the start and end rules for each measurement, and whether the reported values are means, medians, or single observations. At minimum, the authors should include a methodological table indicating, for each indicator, the unit of analysis, measurement procedure, number of observations, summary statistic, dispersion where applicable, improvement formula, and any exclusion or data-cleaning criteria.

 A third critical issue is the technical reproducibility of the software. The manuscript mentions PHP 8.1, Bootstrap, MySQL, TCPDF, and ApexCharts, and it presents logical and physical database designs. However, this information does not allow other researchers or institutions to independently reproduce the installation, configuration, deployment, and use of the system. The authors should add minimal technical documentation including the general system architecture, the exact repository version or commit used in the study, dependencies, installation steps, server configuration, database creation, SQL script or schema, environment variables, anonymized seed or test data, execution flow for the main use cases, and steps to reproduce the reported outputs. This request does not require turning the article into an extensive technical manual; it requires providing the minimum information needed for the tool to be auditable and reusable.

 A fourth critical point concerns the proportionality between results, discussion, and conclusions. The evidence presented supports a bounded claim: the tool reduced the time required for selected academic management processes in one specific institution. However, expressions such as “educational quality”, “digital empowerment”, and “transformative potential” suggest a broader impact that was not directly measured. The study does not assess learning outcomes, user satisfaction, error reduction, sustained adoption, pedagogical quality, or longitudinal institutional impact. Therefore, the authors should moderate the title, abstract, and conclusions, or explicitly define “educational quality” in this article as administrative academic management efficiency. Without additional evidence, it is not methodologically safe to claim broad educational improvement.

 A fifth essential revision concerns the ethics statement. The manuscript states that ethics and consent are not required; however, the study involves institutional processes, academic records, teachers, administrators, and potentially student data. Although the screenshots appear to anonymize names, the authors should clarify whether the data used were real, anonymized, or synthetic; whether institutional authorization was obtained; whether teachers or administrative staff participated in the tests; whether human performance times were measured; and whether an ethics body or institutional authority granted approval, exemption, or a determination of non-applicability. This clarification is necessary because the system manages educational information associated with students.

 In addition to these critical points, I recommend strengthening the traceability between requirements, functionalities, and tests. Table 2 identifies the needs of teachers, the vice principal, and the principal, but it does not connect them through requirement codes to modules, use cases, validation rules, or acceptance criteria. A concise traceability matrix would show which requirement was implemented in which module, how it was validated, and what evidence demonstrates its functionality. It would also be advisable to standardize the grading scale, since the manuscript refers to codes such as AD, A, B, C, SN, NT, and NST without a sufficiently uniform explanation of their differences and conditions of use.

 The technical figures are relevant, but some of them are not sufficiently legible for auditing relationships, entities, or flows. In particular, the database diagrams should be complemented with a schema table listing the main entities, fields, data types, primary keys, foreign keys, and relationships. The use-case screenshots help visualize the interface, but the manuscript should also explain validation rules, role-based permissions, error messages, and exception conditions.

 The discussion could be strengthened if, beyond comparing results with previous studies, it explained the mechanisms through which the tool produced the reported time reductions: data centralization, automated report generation, elimination of manual transcription, reuse of information by academic period, and reduction of administrative steps. The manuscript should also include an explicit limitations section addressing the single-institution design, absence of a control group, possible user learning effects, dependence on connectivity, and lack of direct measurement of errors, satisfaction, or sustained adoption.

 Regarding minor issues, the authors should standardize language, acronyms, and terminology. For instance, Table 1 includes “Análisis y diseño” in Spanish within an English manuscript. The indicators are also named inconsistently: GRT, GST, and RCGT are used in some parts, whereas TRN, TBN, and TELN appear in the conclusions. This variation makes it more difficult to connect objectives, indicators, results, and conclusions. Expressions such as “List of coincidences”, “no editable”, and “bimonthly” should also be revised to improve the precision of the technical English.

 The manuscript presents a valuable, applied contribution and a potentially useful tool for improving the efficiency of school administrative processes. I do not consider that the article should be rejected, since the problem is well justified, the tool exists, the code and data are declared available, and the results are plausible. However, to make the article scientifically sound, the authors must correct the numerical inconsistency, document the measurement procedure more transparently, strengthen the technical reproducibility of the software, justify the ethical handling of educational data, and moderate the conclusions according to the actual scope of the evidence. My editorial recommendation is Approved with reservations.

Are the conclusions about the tool and its performance adequately supported by the findings presented in the article?

Partly

Is the rationale for developing the new software tool clearly explained?

Yes

Is the description of the software tool technically sound?

Partly

Are sufficient details of the code, methods and analysis (if applicable) provided to allow replication of the software development and its use by others?

Partly

Is sufficient information provided to allow interpretation of the expected output datasets and any results generated using the tool?

Partly

Reviewer Expertise:

My areas of expertise are educational technology, software engineering applied to education, web-based information systems, academic management platforms, educational data management, research methodology, and quantitative evaluation of educational software tools. I am particularly able to assess the methodological coherence, technical reproducibility, data interpretation, and proportionality of the conclusions of this article.

I confirm that I have read this submission and believe that I have an appropriate level of expertise to confirm that it is of an acceptable scientific standard, however I have significant reservations, as outlined above.

# Reviewer report: Reviewer response for version 1

**DOI:** 10.5256/f1000research.197001.r480914

**Published:** 2026-05-11

**Authors:** Minara Yeasmin

**Affiliations:** 1 Education, Aliah University Faculty of Humanities and Social Sciences (Ringgold ID: 308965), Kolkata, West Bengal, India

The study is quite significant to the secondary schools. It will enhance the quality of management process at secondary education. The software developed is very significantly optimizes the grade management in rural the  settings of secondary education. The quantitative pre-experimental results confirm significant time reductions and upgraded information quality, pointing out the transformative potential of digital solutions in strengthening academic management processes in schools.

Are the conclusions about the tool and its performance adequately supported by the findings presented in the article?

Yes

Is the rationale for developing the new software tool clearly explained?

Yes

Is the description of the software tool technically sound?

Yes

Are sufficient details of the code, methods and analysis (if applicable) provided to allow replication of the software development and its use by others?

Yes

Is sufficient information provided to allow interpretation of the expected output datasets and any results generated using the tool?

Yes

Reviewer Expertise:

Mathematics Education, Science Education, School Education, Higher Education, Gender Studies,

I confirm that I have read this submission and believe that I have an appropriate level of expertise to confirm that it is of an acceptable scientific standard.

# Reviewer report: Reviewer response for version 1

**DOI:** 10.5256/f1000research.197001.r469460

**Published:** 2026-03-30

**Authors:** Moses Adeleke ADEOYE

**Affiliations:** 1 Al-Hikmah University, Ilorin, Nigeria

**Required Improvements**

 To make the article **scientifically sound and reproducible**, the authors should:

**Provide detailed technical documentation**

 Include system architecture diagrams

 Provide database schema tables (fields, types, relationships)

 Describe key algorithms and validation logic

**Improve reproducibility of analysis**

 Clearly describe:

 sample size

 data collection procedure

 timing measurement methodology

 Specify whether statistical tests were applied

**Add deployment and usage instructions**

 Step-by-step installation guide

 Configuration details (server, database, dependencies)

 Example workflows for users

**Clarify the relationship between paper and GitHub code**

 Indicate version used in the study

 Provide instructions to reproduce reported results using the code

**Clarity of Output Data and Results Interpretation**

 The main limitation is **insufficient reproducibility within the article itself**, specifically:

 Lack of low-level technical details

 Incomplete description of analytical methodology

 Missing deployment and replication instructions

 These issues do **not invalidate the findings**, but they reduce:

 scientific transparency

 reproducibility

 utility for other researchers and practitioners

**Must Address (Critical for Scientific Soundness)**

 Provide detailed methodology for data collection and analysis

 Include full technical specifications (architecture, database, logic)

 Add reproducible deployment and usage instructions

**Should Address (Enhancement)**

 Include code excerpts or pseudocode for key components

 Provide clearer linkage between results and dataset

 Add discussion of limitations (e.g., single-institution study, lack of control group)

 The article is **methodologically sound and practically relevant**, with well-supported conclusions. However, due to **partial reproducibility**, revisions are required to meet full scientific standards. **Overall assessment: Accept with major revisions (focused on reproducibility and technical transparency).**

Are the conclusions about the tool and its performance adequately supported by the findings presented in the article?

Yes

Is the rationale for developing the new software tool clearly explained?

Yes

Is the description of the software tool technically sound?

Yes

Are sufficient details of the code, methods and analysis (if applicable) provided to allow replication of the software development and its use by others?

Partly

Is sufficient information provided to allow interpretation of the expected output datasets and any results generated using the tool?

Yes

Reviewer Expertise:

Educational Leadership, Research Method

I confirm that I have read this submission and believe that I have an appropriate level of expertise to confirm that it is of an acceptable scientific standard, however I have significant reservations, as outlined above.
